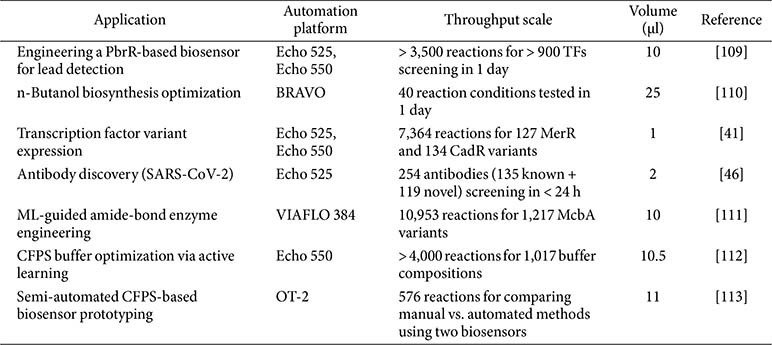# Automated and Programmable Cell-Free Systems for Scalable Synthetic Biology with a Focus on Biofoundry Integration

**DOI:** 10.4014/jmb.2507.07019

**Published:** 2025-09-16

**Authors:** Ji-Su Jun, Sujin Hong, Jun-Hong Park, Jonghyeok Shin, Dae-Hee Lee

**Affiliations:** 1 Synthetic Biology Research Center, Korea Research Institute of Bioscience and Biotechnology (KRIBB), Daejeon 34141, Republic of Korea; 2 Department of Biosystems and Bioengineering, KRIBB School of Biotechnology, University of Science and Technology (UST), Daejeon 34113, Republic of Korea; 3 Korea Biofoundry, Korea Research Institute of Bioscience and Biotechnology (KRIBB), Daejeon 34141, Republic of Korea; 4 Graduate School of Engineering Biology, Korea Advanced Institute of Science and Technology (KAIST), Daejeon 34141, Republic of Korea; 5 Department of Integrative Biotechnology, College of Biotechnology and Bioengineering, Sungkyunkwan University, Suwon 16419, Republic of Korea

**Keywords:** Cell-free protein synthesis, biofoundry, synthetic biology, automation, high-throughput screening, programmable biological engineering

## Abstract

Cell-free protein synthesis (CFPS) has been used as a transformative technology in synthetic biology, providing a programmable, scalable, and automation-compatible platform for biological engineering. Freed from the limitations of cell viability and growth, CFPS enables rapid design iteration, precise control of reaction conditions, and high-throughput experimentation. Recent integration of CFPS with biofoundries-automated, high-throughput biological engineering platforms-has dramatically accelerated the Design-Build-Test-Learn cycle, facilitating applications such as enzyme engineering, metabolic pathway prototyping, biosensor development, and remote biomanufacturing. Advances in automation technologies, including liquid-handling robotics and digital microfluidics, have further enhanced the scalability and reproducibility of CFPS workflows. Additionally, coupling CFPS with machine learning has enabled predictive optimization of genetic constructs and biosynthetic systems. This review highlights the technological innovations driving the convergence of CFPS and automated biofoundries, outlining current capabilities, challenges, and future directions toward programmable, scalable, and distributed biological engineering.

## Introduction

Synthetic biology is a field that combines biology and engineering to design and build new biological systems. Synthetic biology aims to engineer biological functions that are predictable, modular, and scalable—enabling applications in therapeutics, diagnostics, and sustainable biomanufacturing [1-4]. Achieving these goals increasingly depends on platforms that support rapid design iteration, high-throughput experimentation, and improved reproducibility [5]. However, functional prototyping has traditionally relied on cell-based systems, which are often limited by growth dependency, unpredictable gene regulation, and metabolic burden [6, 7]. These limitations delay iteration and hinder scalability, underscoring the need for alternative platforms that enable faster iteration, reduced variability, and compatibility with non-natural inputs and automated systems.

These limitations are particularly pronounced in microbial systems widely used in industrial biotechnology, such as *Escherichia coli* or *Saccharomyces cerevisiae*, where gene expression can be disrupted by feedback regulation, plasmid instability, or protein toxicity. In such hosts, rapid prototyping of complex pathways or difficult-to-express enzymes is constrained by lengthy transformation, growth, and selection cycles. Cell-free protein synthesis (CFPS) platforms address these issues by decoupling gene expression from living cells, enabling immediate access to the transcription–translation machinery without host-dependent interference. This open and tunable environment facilitates faster iteration, supports expression of otherwise toxic proteins, and allows direct manipulation of enzyme concentrations and reaction conditions—features particularly valuable in microbial enzyme engineering and metabolic pathway optimization.

One promising alternative is CFPS, an *in vitro* gene expression platform that enables transcription and translation from cell lysates or purified components, independent of living cells. Its open and tunable environment allows precise control over reaction conditions, rapid expression of toxic proteins, difficult to fold, or incompatible with cellular metabolism, and broad compatibility with various DNA templates [8-10]. Furthermore, CFPS supports a broad range of applications, including protein engineering, enzyme screening, biosensor development, and metabolic pathway prototyping [11-13]. These features position CFPS as a compelling alternative to conventional cell-based expression systems.

Biofoundries are becoming core infrastructure in synthetic biology by integrating automated hardware systems—such as liquid-handling robotics, high-throughput experimentation platforms—with software tools for data management, experimental control, and iterative optimization [5, 14-18]. Within these automated and integrated platforms, CFPS is emerging as a powerful engine, particularly by accelerating the “Test” phase of the Design-Build-Test-Learn (DBTL) cycle. Its compatibility with automation and miniaturized reaction formats enables scalable, parallel experimentation, dramatically increasing throughput and reducing iteration time [17, 19, 20].

In this review, we highlight recent advances that are transforming CFPS into a core platform for synthetic biology. We focus on its integration with automation, high-throughput experimentation, and programmable workflows. These innovations enable rapid prototyping, support remote biomanufacturing, and accelerate intelligent biological design. Together, they mark a shift from traditional cell-based systems toward programmable, scalable, and automated bioengineering.

## Overview of CFPS Research

### Brief History of CFPS

The concept of CFPS dates to the mid-20th century, when foundational studies demonstrated that biological macromolecule could be synthesized outside living cells. Early experiments by Nirenberg and Matthaei employed *E. coli* lysates to decipher the genetic code, establishing the fundamental utility of *in vitro* translation systems [21]. Subsequently, the development of more refined lysate preparation methods, including the use of S30 extracts from *E. coli*, enabled broader applications of CFPS systems for protein synthesis [22]. Throughout the 1970s to 1990s, significant advances were made in optimizing CFPS yields by improving energy regeneration systems, adjusting ionic conditions, and stabilizing reaction components [23-25]. The introduction of continuous-exchange cell-free (CECF) systems further enhanced reaction longevity and protein yield, laying the groundwork for large-scale applications [26]. Entering the 21^st^ century, cell-free platforms diversified beyond prokaryotic systems. Lysates derived from wheat germ [27, 28], yeast [29, 30], insects [31, 32], rabbit reticulocytes [33], Chinese hamster ovary (CHO) cells [34], HeLa cells [35], and more recently, hybrid host systems [36-38], have expanded the versatility of CFPS for expressing a wide range of proteins, including membrane proteins and complex eukaryotic proteins. More recently, advances in synthetic biology, automation, and freeze-drying technologies have dramatically expanded the utility of CFPS platforms [39-41]. Today, cell-free systems are recognized not only as tools for fundamental research but also as key technologies in areas such as on-demand biomanufacturing, point-of-care diagnostics, synthetic pathway prototyping, and extraterrestrial bioproduction [42-47].

### Components of CFPS

**Fig. 1:**
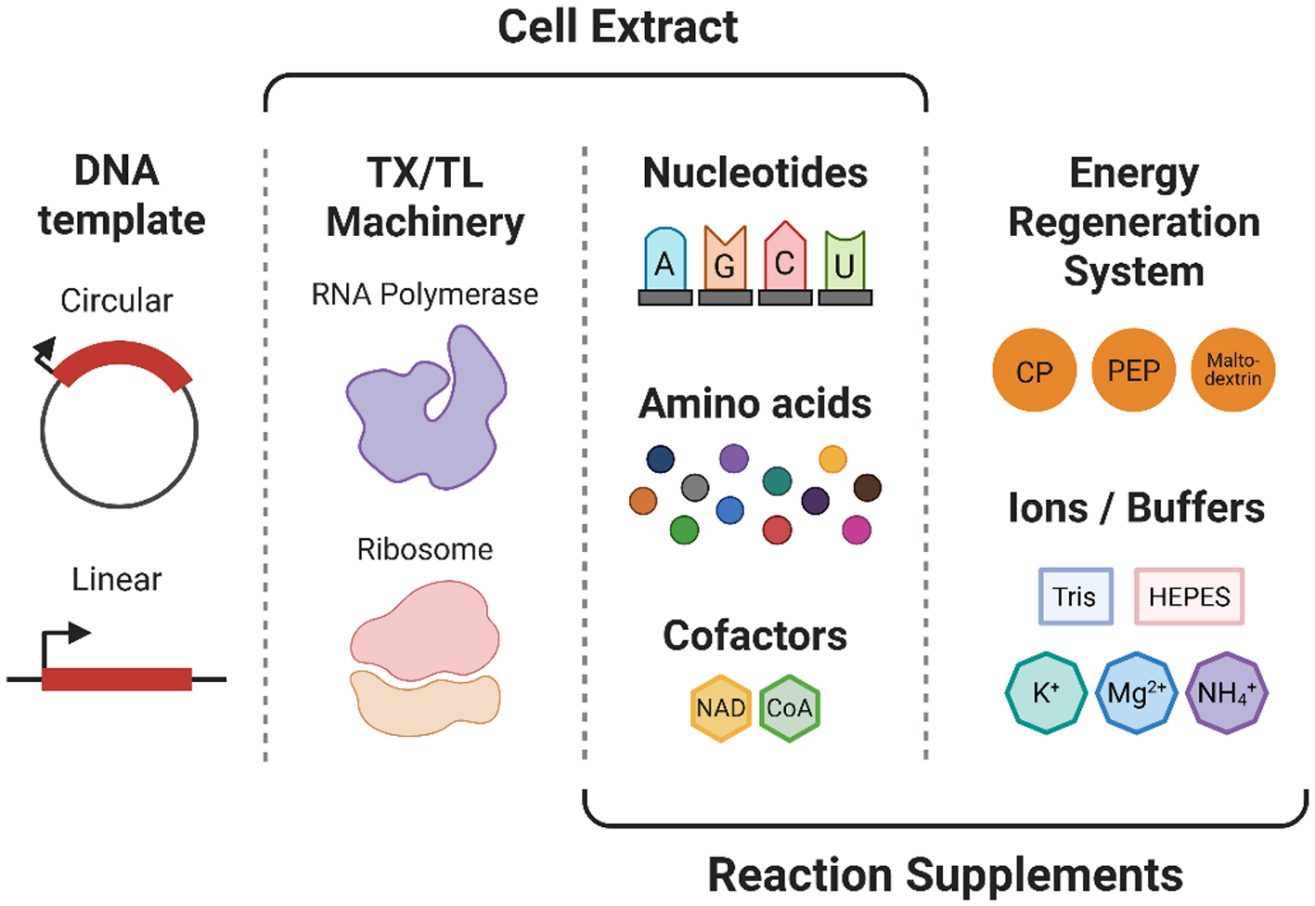
Major components of cell-free protein synthesis (CFPS). A CFPS reaction consists of DNA templates, transcription (TX)-translation (TL) machineries, nucleotides, amino acids, energy regeneration systems, and cofactors/buffers. These elements work together in cell-free environments to drive gene expression independent of living cells.

A functional CFPS system consists of several essential components that collectively enable efficient transcription and translation outside living cells (Fig. 1). First, the DNA template provides the genetic blueprint, typically supplied as plasmid DNA, linear PCR products, or synthetic oligonucleotides [48]. Optimization of promoter strength, untranslated regions, and codon usage is critical for effective protein synthesis. Second, the transcription (TX) and translation (TL) machinery orchestrates gene expression. RNA polymerase (RNAP), either endogenous or phage-derived (*e.g.*, T7 polymerase) [49, 50], initiates transcription, supported by nucleoside triphosphates (NTPs) and, when needed, additional regulatory elements. Ribosomes and translation factors, along with tRNAs and amino acids, are typically supplied through the lysate, although supplementation is sometimes required, particularly for systems incorporating non-canonical amino acids [51]. Third, an energy regeneration system is crucial to maintain ATP and GTP levels. Systems based on phosphoenolpyruvate (PEP) [52], creatine phosphate [26], or more recently, maltodextrin-based systems [52, 53] are commonly used to prolong reaction longevity. Fourth, cofactors, ions, and buffer components maintain reaction stability. Supplements such as NAD^+^, CoA [52], magnesium ions (Mg^2+^), potassium ions (K^+^), and buffering agents like HEPES ensure optimal biochemical conditions. Crowding agents and polyamines further enhance protein yields by mimicking the cytoplasmic environment. Among these, most components—including TX/TL machinery, cofactors, and crowding agents—are naturally provided by crude cell lysates. Commonly used sources such as *E. coli*, wheat germ, rabbit reticulocytes, and insect cells, with lysate preparation methods significantly influencing extract quality and performance. In addition to crude extracts, fully reconstituted systems such as the Protein synthesis Using Recombinant Elements (PURE) system have been developed [54]. Comprising individually purified ribosomes, tRNAs, translation factors, and RNAP, the PURE system offers high compositional control and reduced background activity, facilitating high-fidelity protein synthesis [55]. However, its scalability and cost remain limiting factors compared to lysate-based approaches. Together, these components form a modular biochemical environment capable of executing gene expression programs independently of cellular contexts, underpinning a wide range of emerging applications in synthetic biology.

### General Application of CFPS

**Fig. 2:**
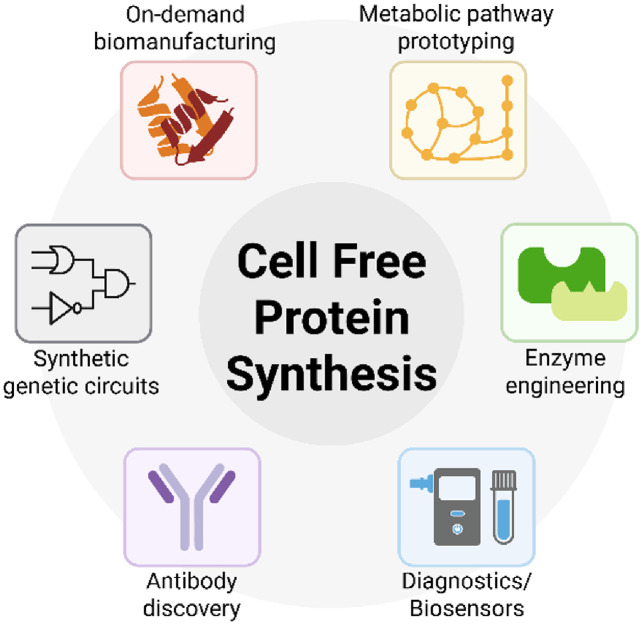
General applications of CFPS. CFPS supports a broad range of applications, including metabolic pathway prototyping, enzyme engineering, antibody screening, genetic circuit testing, and biosensor development. Its open and tunable environment enables rapid and flexible prototyping across synthetic biology, diagnostics, and biomanufacturing.

CFPS is increasingly being used as a flexible platform across metabolic engineering, therapeutic protein production, synthetic biology, and field-deployable diagnostics (Fig. 2). Notably, many of these applications involve microbial enzymes, transcriptional regulators, or metabolic pathways originally derived from bacteria and yeast. By enabling rapid, *in vitro* testing of these microbial components—often without the need for transformation or cultivation—CFPS offers a significant acceleration over traditional strain engineering workflows. This is especially valuable in microbial biotechnology, where iterative pathway optimization and toxic gene expression often pose bottlenecks.

One of its most well-established applications is in metabolic pathway prototyping, where CFPS allows for rapid *in vitro* reconstitution and testing of multi-enzyme cascades, such as those producing mevalonate, 1,4-butanediol, or styrene [56-59]. These systems enable quantitative analyses of flux, enzyme ratios, and cofactor dynamics [60-63], facilitating the rational design and optimization of biosynthetic pathways. Unlike traditional cell-based systems, CFPS allows direct control over enzyme concentrations, cofactor levels, and reaction conditions, facilitating fine-tuned optimization of complex metabolic networks [20, 62, 64].

Beyond pathway construction, CFPS plays a pivotal role in enzyme engineering. It supports high-throughput screening of enzyme variants, active-site mutants, and cofactor dependencies [65-70]. This approach is particularly useful when working with toxic enzymes or labile intermediates that are difficult to handle in living systems, making CFPS a powerful tool for biocatalyst discovery and optimization. CFPS has also enabled rapid expression and screening of antibodies, including IgGs, scFvs, nanobodies, and cytokines, using oxidizing extracts and engineered folding environments [71-80]. These systems have been applied to both affinity screening and functional assays such as neutralization and receptor blocking, significantly shortening the antibody discovery timeline. In the field of synthetic biology, CFPS has accelerated the prototyping of genetic circuits that regulate gene expression in response to defined inputs. These circuits enable programmable responses such as decision-making logic, inducible control, and therapeutic gene regulation [75, 81-86], and provide a cell-free platform for testing network dynamics before cellular implementation. One of the most impactful applications is the development of biosensors. Modular CFPS-based sensors utilizing toehold switches, aptamers, or transcription factors have been used to detect viral RNA, small molecules, and environmental toxins [87-95]. These reactions can be freeze-dried and embedded into portable paper or microfluidic formats for on-site diagnostics [96-101]. Furthermore, lyophilized CFPS kits allow on-demand protein production in low-resource or remote settings, including clinical, military, and even spaceflight scenarios [42, 44, 102, 103]. These features highlight the distributable and programmable potential of CFPS prior to full automation, as discussed in Sections 2.2.3 and 2.2.4.

## Application of CFPS with Automation

### Automation of High-Throughput Assays Using CFPS

High-throughput CFPS workflows typically utilize 96-, 384-, or 1536-well plate formats in combination with robotic liquid handling systems to ensure reproducibility and precision. Automated platforms such as the Opentrons OT-2 and Labcyte Echo provide accurate and consistent low-volume dispensing [104-107]. This is particularly advantageous in CFPS, where small reaction volumes and costly reagents demand tight control. The modular and miniaturized nature of CFPS is inherently well-suited for integration with liquid-handling robotics, enabling parallel execution of hundreds to thousands of reactions with minimal variability. The examples listed in Table 1 show how CFPS platforms, when paired with high-precision liquid handlers, enable scalable and field-adaptable molecular workflows within and beyond biofoundry settings. This compatibility enhances throughput, reduces manual error, and facilitates the standardized implementation of complex experimental designs—making CFPS a core component of high-throughput synthetic biology pipelines [108].

CFPS platforms allow systematic and precise control over *in vitro* gene expression, including the number, order, and concentration of genes. This high degree of tunability supports highly programmable experimental designs. For example, Sawasaki *et al*. demonstrated the simultaneous expression of over 50 proteins using an *E. coli* extract-based CFPS system, highlighting its potential for high-throughput applications [12]. Building on this, Dudley *et al*. integrated automated Golden Gate assembly with CFPS to screen hundreds of genetic constructs in parallel, showcasing the power of combining systematic gene assembly and expression control in a single workflow [17]. Beyond protein production, parallelized CFPS has been applied to enzyme activity screening and genetic circuit evaluation. Ekas *et al*. engineered over 7,000 MerR and CadR transcription factor variants using an acoustic liquid-handling-assisted CFPS pipeline, completed in under 48 h [41]. Similarly, Hunt *et al*. developed a fully automated antibody discovery platform combining cell-free DNA assembly, sdFab expression, and AlphaLISA assays. This system screened over 250 antibody candidates, including EUA-approved SARS-CoV-2 neutralizers, within just 24 h [46]. Automated DNA assembly, cell-free expression, and high-throughput assays—often powered by liquid-handling machinery—are now central to the DBTL cycle in modern biofoundries. These workflows enable parallel assembly, expression, and screening, generating data that informs machine learning–guided design. By supporting scalable, data-driven iteration, automated CFPS platforms enhance the speed, precision, and reproducibility of biological engineering. To further expand these capabilities, integration-friendly hardware, standardized protocols, and robust data-sharing frameworks will be essential.

### Machine Learning–Guided Optimization Enabled by CFPS

The integration of CFPS with machine learning (ML) has emerged as a powerful framework for accelerating protein engineering [114], enzyme optimization [111], and antimicrobial peptide (AMP) design [115]. CFPS provides rapid prototyping, enabling efficient generation and testing of genetic designs. Its ability to support fast, scalable, and automated experimentation makes it particularly well-suited for producing the large, high-quality datasets needed to train predictive ML models. Most supervised learning approaches require hundreds to thousands of experimental datapoints, yet collecting such datasets in cell-based systems is often slow and labor-intensive [116-118]. CFPS addresses this challenge by enabling rapid, parallelized, and repeatable experiments, particularly when combined with liquid handling automation. Borkowski *et al*. demonstrated this synergy by optimizing CFPS buffer compositions using active learning. Eleven reaction components were varied across four concentrations, yielding over 4 million combinations. Remarkably, after testing only ~1,000 compositions, they achieved a maximum 34-fold increase in protein yield with a predictive accuracy (R²) of 0.93, using as few as 20 data points to generalize the model to new lysates [112]. Pandi *et al*. applied a generative ML model to design novel AMPs. Approximately 500,000 peptide sequences were computationally generated, and 500 candidates with high predicted activity were selected and expressed using CFPS. Among these, 30 showed antimicrobial activity and six exhibited broad-spectrum efficacy with low cytotoxicity [115]. The use of CFPS allowed rapid prototyping without concerns about host toxicity, highlighting its utility in early-stage therapeutic development. Landwehr *et al*. developed a sequence-to-function ML model for amide-bond forming enzymes. A library of 1,217 enzyme variants was expressed using CFPS and screened across 10,953 reactions. A ridge regression model trained on this dataset predicted high-performance variants with up to 42-fold improvements in catalytic activity. This workflow was fully automated within a biofoundry pipeline, including DNA assembly, expression, screening, ML modeling, and redesign [111]. Thornton *et al*. employed CFPS to enable real-time ML-guided optimization of the synthetic protease Con1. A library of 192 variants was expressed and tested using a FRET-based activity assay. These data trained the Active Learning–assisted Directed Evolution (ALDE) framework, which proposed 32 additional variants. Several achieved up to 4-fold improvements in activity. Each experimental round—from expression to functional measurement—was completed in under 6 h [114]. Together, these studies demonstrate that CFPS is not only compatible with ML-driven workflows but also structurally advantageous. Platforms like Echo and OT-2 facilitate hundreds to thousands of reactions daily with high consistency and minimal human intervention. CFPS thus serves as a data-generation engine for predictive models, accelerating biological design through closed-loop iteration. As CFPS–ML integration matures, it is expected to expand into more complex applications, including metabolic pathway optimization, antibody engineering, biosensor tuning, and diagnostics. With ongoing advances in automation, miniaturization, and real-time analytics, CFPS is evolving from a prototyping tool into a foundational platform for data-driven synthetic biology.

### Automated CFPS for Biosensor Development

CFPS has enabled the development of highly modular biosensors free from the constraints of cellular viability. These sensors offer fast response, ease of customization, and strong potential for field-deployable diagnostics [119]. When combined with automated liquid-handling systems and standardized workflows, cell-free biosensors can be manufactured, deployed, and operated with minimal human intervention, further broadening their applicability in synthetic biology and point-of-care settings. One prominent example is ROSALIND (RNA Output Sensors Activated by Ligand Induction) platform, which uses *in vitro* transcription reactions regulated by allosteric transcription factors to detect environmental contaminants such as metals, antibiotics, and small molecules. ROSALIND’s design, based on freeze-dried components, allows sensors to be prepared in advance, stored at room temperature, and activated upon simple rehydration with the target sample. Moreover, the outputs are fluorescent RNA aptamers, enabling rapid signal readout without the need for complex protein translation machinery [120]. Automation has been critical in scaling the production and deployment of such biosensors. For instance, Brown *et al*. implemented a semi-automated pipeline for constructing and screening transcriptional biosensors targeting water contaminants, showcasing how robotic liquid handling can dramatically improve throughput and consistency [113]. Similarly, automated systems such as Echo 525 enable highly precise, tipless transfer of nanoliter-scale reaction volumes, optimizing reagent use and minimizing human error [121]. Building on these trends, Kim *et al*. developed a cell-free transcription factor-based biosensor for detecting cellobiose, using an acoustic liquid handling system to automate the preparation and screening of cellobiohydrolases targeting insoluble substrates. The cell-free-based biosensor showed higher sensitivity than conventional assays and enabled miniaturized reactions (~1.56 μl) with high reproducibility, significantly reducing reagent consumption [121]. Importantly, recent global health and environmental crises have highlighted the urgent need for rapid and adaptable biosensor development [122]. In this context, CFPS-based biosensors have been successfully applied to detect a wide range of targets including viral and bacterial pathogens, small molecule drugs, endocrine disrupting chemicals, and protein therapeutics across diverse sample types such as sputum, feces, nasal secretions, urine, and blood [87, 92, 123-125]. While direct examples of CFPS-discovered biosensors being applied in microbial strain development are currently limited, their potential in this domain is increasingly recognized. The rapid response and modularity of transcription factor–based CFPS biosensors make them particularly well suited for upstream screening of enzyme activity and pathway intermediates—critical steps that inform downstream strain optimization. As CFPS platforms continue to integrate with automated workflows and machine learning, their role in accelerating design–build–test cycles for strain engineering is expected to expand significantly.

### Extending Biofoundry Capabilities to Remote and Extreme Environments

While current synthetic biology and biofoundries primarily operate in centralized laboratories equipped with sophisticated instrumentation and trained personnel, there is growing interest in extending their capabilities to remote, resource-limited, or extreme environments. In these contexts, CFPS—particularly in freeze-dried, programmable, and automation-compatible formats—has emerged as a key enabler of distributed biodesign. CFPS platforms can serve as deployment-ready outputs of biofoundry workflows, allowing pre-designed genetic programs to be executed outside traditional lab settings. This capability supports decentralized biomanufacturing, point-of-care diagnostics, and biosensing in diverse environments—from rural clinics to space missions—thereby extending the functional boundaries of synthetic biology beyond the laboratory. Freeze-dried cell-free systems (FD-CFS) offer utility due to their ambient-temperature stability and ease of rehydration. Kocalar *et al*. successfully demonstrated FD-CFS protein synthesis aboard the International Space Station (ISS), confirming that genetic expression is feasible under microgravity condition [42]. When coupled with pre-programmed automation, such systems can be operated by non-specialists, including astronauts, to perform complex molecular tasks. Recent developments have further advanced the portability and accessibility of CFPS workflows. Didovyk *et al*. introduced an autolysis-based lysate preparation protocol requiring only freeze-thaw or freeze-dry and rehydration steps, eliminating the need for specialized equipment such as high pressure homogenizers, sonicators, ultracentrifuges, and dialysis equipment [126]. This approach enables on-site preparation of CFPS-ready lysates, supporting applications such as colorimetric heavy metal detection in the field. In addition, remote control of molecular operations has been enabled by digital microfluidic (DMF) platforms. Liu *et al*. developed a programmable DMF system capable of manipulating individual droplets containing CFPS components, facilitating real-time remote execution of reactions [127]. Such systems offer increased flexibility and automation, particularly valuable in settings where physical access is limited. These advances align with the broader concept of resilient biology, which aims to engineer biological systems capable of robust function under unpredictable and extreme conditions. Brooks and Alper emphasized that moving synthetic biology beyond laboratory settings will require modular, lyophilized systems combined with autonomous control and minimal infrastructure [43]. They highlighted the importance of robust, decentralized platforms for on-demand vaccine production, biosurveillance, and point-of-care diagnostics. While most CFPS efforts have focused on protein synthesis, related non-cellular molecular technologies have also been applied in point-of-care diagnostics. Arjuna *et al*. developed a cell free isothermal amplification system (CF-LAMP) for detecting EGFR mutations in plasma cell-free DNA with high specificity and minimal instrumentation [128]. Though not a classical CFPS system, it shares the goal of enabling instrument-light molecular diagnostics outside traditional lab environments. Unlike conventional PCR-based approaches, CF-LAMP operates without the need for thermal cyclers or other specialized instrumentation, making it highly suitable for point-of-care use in resource-limited settings. Collectively, these developments illustrate how CFPS platforms—particularly when combined with freeze-drying and remote-controllable microfluidic systems—are enabling a new paradigm of distributed biodesign. Here, “distributed” refers not only to the geographical separation from centralized laboratories but also to the democratization of access, empowering non-specialists to execute sophisticated molecular programs in low-resource or extreme environments. Rather than replacing biofoundries, these systems extend their operational footprint by serving as deployment-ready, execution-stage platforms. Freeze-dried and remotely operable CFPS kits act as the “last-mile” of the synthetic biology pipeline, enabling programmable and scalable biological functions to be delivered and run far beyond the lab—realizing the vision of biofoundry-enabled, distributed biological engineering.

### Sustained and Autonomous Operation of Biofoundry-Enabled CFPS

To fully realize the potential of CFPS in automated and field-deployable systems, long-term operability is essential. Sustained reactions are particularly critical in continuous biomanufacturing workflows and autonomous biofoundry platforms, where stability, resource efficiency, and minimal intervention are key. Recent advances in system design, energy regeneration, and biochemical stabilization have significantly extended CFPS duration, opening new opportunities for long-term and hands-free applications [129, 130]. A foundational approach is the CECF system, in which a reaction chamber is separated from a feeding chamber by a semi-permeable membrane. This configuration allows continuous substrate replenishment and byproduct removal. Sawasaki *et al*. first demonstrated this method using a dialysis-based system, achieving protein synthesis for up to 14 days [12]. The concept of continuous flow of cell-free protein synthesis dates back to the 1980s [26]. Prolonged CFPS reactions also depend on the source and quality of lysates. CHO-based CECF systems have achieved sustained expression of complex proteins—including membrane proteins and antibody fragments—for 48 h or longer, with yields up to 980 μg/ml [34]. Insect cell lysates are also being explored for their enhanced folding and enzymatic stability, especially for eukaryotic proteins requiring post-translational modifications [131]. To support longer reaction durations, various substrate replenishment and energy regeneration strategies have been developed. Kim and Swartz demonstrated ATP regeneration using periodic additions of glycolytic intermediates and creatine phosphate [52]. Calhoun and Swartz further optimized this with a cost-effective system using glucose and nucleoside monophosphates [12]. Additionally, Yamane *et al*. introduced selective amino acid and energy molecule replenishment to reduce resource depletion and extend reaction longevity [132]. These approaches are especially useful in batch-mode CFPS, where substrate depletion and byproduct accumulation limit the reaction duration. Beyond biochemical stabilization, physical approaches have also emerged. Jewett and Swartz mimicked intracellular crowding and ionic conditions to enhance translation machinery stability [8]. Building on this, Ouyang *et al*. developed a hydrogel encapsulation method, where transcription–translation components are immobilized within polymer networks. This enabled protein synthesis to persist for over 28 days, offering a robust framework for ultra-long-duration CFPS, especially in modular and field-deployable systems [133]. Scaling CFPS without sacrificing productivity has further improved long-term applications. Zawada *et al*. successfully scaled cytokine production from microscale to manufacturing-scale without loss of yield [75]. Zhou *et al*. addressed oxygen and byproduct limitations with a tube-in-tube reactor design that enabled continuous flow, optimized residence time, and protein yields up to 3 mg/ml [134]. These innovations enhance the scalability and engineering flexibility of CFPS, reinforcing its suitability for integration into automated biofoundries. Persistent challenges remain, including enzyme degradation, RNase contamination, and energy depletion. While ATP regeneration strategies reduce reliance on high-energy substrates, scalability and cost-efficiency remain concerns. Future directions include integrating cofactor recycling (*e.g.*, NAD^+^, FMN) and modular energy modules to further extend CFPS duration. As biochemical and engineering advances continue to mature, CFPS systems are increasingly being integrated with automation to support scalable, hands-free operations. Long-term, continuous CFPS is emerging as a key enabler of modern biomanufacturing—mimicking traditional fermentation while reducing waste and enabling applications in decentralized, on-demand production. These strategies not only enhance batch-mode workflows but also lay the foundation for autonomous, around-the-clock biofoundry operations. By combining prolonged operability with programmable automation, CFPS becomes a core driver of self-optimizing, intelligent biological design and manufacturing.

## Conclusion and Future Directions

**Fig. 3:**
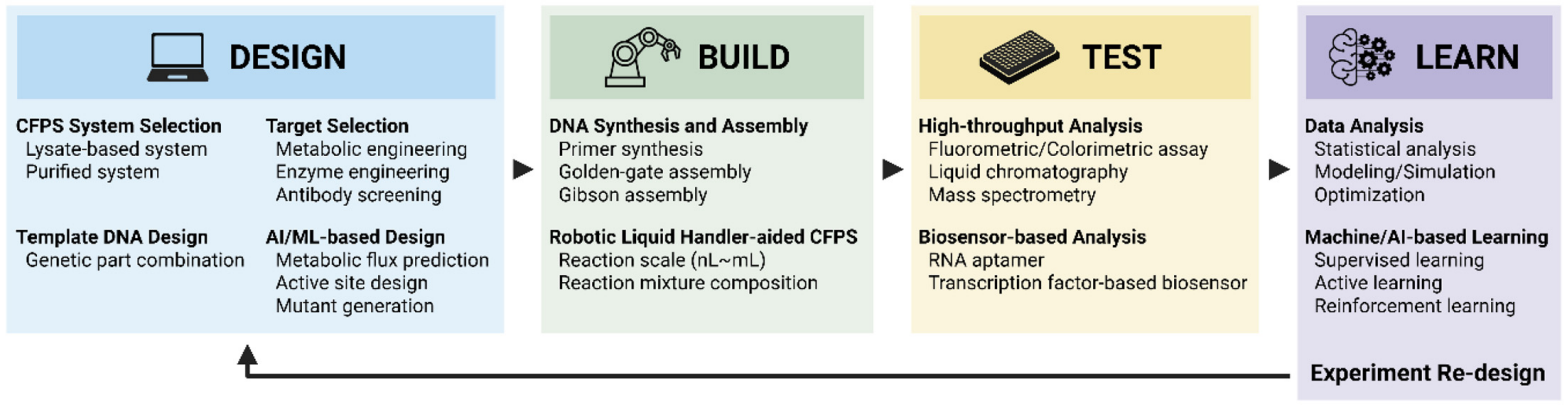
Integration of CFPS into the Design-Build-Test-Learn (DBTL) cycle. CFPS enables rapid prototyping and high throughput testing within the DBTL workflow by decoupling gene expression from living cells. Its compatibility with automation, programmability, and reproducibility accelerates synthetic biology research and biofoundry operations.

CFPS has evolved from a protein expression tool into a key component of modern biofoundries. Its programmability, scalability, and compatibility with automation make it ideally suited to support the DBTL framework that underpins synthetic biology today (Fig. 3). Within the automated DBTL workflow, CFPS enables rapid, high-throughput, and standardized testing of genetic constructs, significantly accelerating the experimental cycle and reducing development timelines [6, 7, 9]. Free from cellular growth constraints and metabolic complexity, CFPS allows for precise control of reaction conditions and is ideally suited for small-volume, high-throughput, and repeatable workflows [9, 15]. Notably, CFPS’s open reaction environment and high reproducibility make it highly compatible with automation tools such as liquid-handling robots and DMFs, enabling thousands of parallel reactions to be executed with high precision [41, 108]. As a result, CFPS has moved beyond being merely a protein expression tool and is increasingly regarded as a core automation-ready platform within biofoundries [17, 19, 20].

Importantly, recent studies have shown that CFPS-based prototyping can successfully inform *in vivo* design, further enhancing its translational relevance. For instance, Landwehr *et al*. applied deep learning to CFPS-derived data to engineer amide-bond forming enzymes, with several variants demonstrating up to 42-fold improvement in activity when expressed in *E. coli* [111]. Likewise, Karim *et al*. used CFPS to optimize enzymes and pathway configurations for the biosynthesis of monoterpenes and butanediol [47, 60]. Their CFPS-based findings were successfully translated into microbial hosts including *E. coli* and *S. cerevisiae*, leading to enhanced *in vivo* production. These cases underscore the growing capacity of CFPS to bridge *in vitro* screening with functional performance in living systems.

Despite these advantages, several technical bottlenecks still limit the broader adoption and industrial scaling of CFPS platforms. One persistent challenge is the variability in lysate quality across batches, which can significantly affect protein yield and reproducibility. Additionally, many lysate-based systems lack robust post-translational modification machinery, constraining the expression of eukaryotic proteins that require glycosylation or disulfide bond formation. Reaction longevity remains another limitation, particularly in batch-mode formats, where energy sources are rapidly depleted and byproducts accumulate [7, 133]. Moreover, the cost and complexity of preparing high-quality lysates or PURE systems can restrict scalability and accessibility in resource-limited settings. Addressing these bottlenecks will require continued innovation in extract stabilization, cofactor recycling, and system standardization. Several earlier studies have proposed solutions to these challenges. For instance, energy regeneration systems [52], simplified lysate preparation [126], and stabilized CFPS systems [135] have improved cost-efficiency and reaction longevity. Lysate improvement [136] and cofactor recycling strategies [137] further enhance system performance, while scalable batch-mode platform [138] and paper-based platform [92] support industrial deployment. These advances collectively highlight practical paths toward more robust and scalable CFPS platforms.

These features naturally align with the demands of automated synthetic biology research cycles, enabling CFPS to serve as a practical experimental backbone for diverse applications such as enzyme engineering, biosensor development, molecular diagnostics, and remote biomanufacturing [9, 11, 42]. To address these issues, strategies such as continuous exchange systems [26], ATP regeneration methods [52, 132], and component immobilization techniques [133] are being actively explored. The future development of CFPS will advance along several key directions, particularly in combination with automation. First, integration with machine learning and real-time feedback systems is expected to dramatically improve the accuracy and speed of CFPS-based experimental design [111, 112, 116]. Second, to enhance compatibility and scalability across biofoundries, standardization of reagents, protocols, and data formats will be essential. Such standardization will improve reproducibility and facilitate cross-institutional collaboration and technology transfer [14, 15]. Third, while most current CFPS platforms are based on *E. coli*-derived prokaryotic extracts, expanding to eukaryotic systems is essential for expressing complex proteins. The advancement of eukaryotic CFPS, in tandem with automation technologies, will be a critical turning point for efficient and precise production of high-value proteins such as therapeutic enzymes, antibodies, and extracellular signaling factors [6, 8, 40, 139, 140]. Lastly, the emergence of freeze-dried, field-deployable CFPS systems—combined with long-duration, hands-free operation capabilities—paves the way for distributed and autonomous biofoundries. These platforms can execute pre-designed workflows beyond conventional laboratories, enabling real-time biomanufacturing, biosensing, and diagnostics in resource-limited, remote, or even extraterrestrial settings. Such developments signify a paradigm shift from centralized automation toward programmable, scalable, and geographically decoupled biological design and execution [42, 44, 126, 127].

In summary, CFPS has matured into a versatile and automation-compatible technology that plays a central role in modern biofoundries. Its rapid, modular, and programmable nature allows researchers to decouple biological design from traditional cell-based constraints, enabling faster iteration, higher throughput, and greater reproducibility. Through integration with standardized hardware, data-driven optimization tools, and field-deployable formats, CFPS is redefining how biological functions are designed, built, and tested across diverse environments.

## Acknowledgments

This work was supported by the Bio & Medical Technology Development Program of the National Research Foundation of Korea (Grant numbers: RS-2024-00445145, RS-2024-00509115, RS-2024-00398252), funded by the Korean government (MSIT) and the KRIBB Research Initiative Program (grant number KGM1302511). Additional support was provided by the Korea-US Collaborative Research Fund (KUCRF), funded by the Ministry of Science and ICT and the Ministry of Health & Welfare, Republic of Korea (Grant number: RS-2024-00468410).

## Figures and Tables

**Table 1:** Examples of automated liquid handling for CFPS.